# Association between Walking Speed and Age in Healthy, Free-Living Individuals Using Mobile Accelerometry—A Cross-Sectional Study

**DOI:** 10.1371/journal.pone.0023299

**Published:** 2011-08-10

**Authors:** Michaela Schimpl, Carmel Moore, Christian Lederer, Anneke Neuhaus, Jennifer Sambrook, John Danesh, Willem Ouwehand, Martin Daumer

**Affiliations:** 1 Sylvia Lawry Centre for Multiple Sclerosis Research e.V. – The Human Motion Institute, Munich, Germany; 2 Trium Analysis Online GmbH, Munich, Germany; 3 Department of Public Health and Primary Care, University of Cambridge, Cambridge, United Kingdom; 4 Department of Haematology, University of Cambridge and NHS Blood and Transplant, Cambridge, United Kingdom; Universidad Europea de Madrid, Spain

## Abstract

**Context:**

Walking speed is a fundamental parameter of human motion and is increasingly considered as an important indicator of individuals' health status.

**Objective:**

To evaluate the relationship of gait parameters, and demographic and physical characteristics in healthy men and women.

**Design, Setting, and Participants:**

Recruitment of a subsample (*n* = 358) of male and female blood donors taking part in the Cambridge CardioResource study. Collection of demographic data, measurement of physical characteristics (height, weight and blood pressure) and assessment of 7-day, free-living activity parameters using accelerometry and a novel algorithm to measure walking speed. Participants were a median (interquartile range[IQR]) age of 49 (16) years; 45% women; and had a median (IQR) BMI of 26 (5.4).

**Main Outcome Measure:**

Walking speed.

**Results:**

In this study, the hypothesis that walking speed declines with age was generated using an initial ‘open’ dataset. This was subsequently validated in a separate ‘closed’ dataset that showed a decrease of walking speed of −0.0037 m/s per year. This is equivalent to a difference of 1.2 minutes, when walking a distance of 1 km aged 20 compared to 60 years. Associations between walking speed and other participant characteristics (i.e. gender, BMI and blood pressure) were non-significant. BMI was negatively correlated with the number of walking and running steps and longest non-stop distance.

**Conclusion:**

This is the first study using accelerometry which shows an association between walking speed and age in free-living, healthy individuals. Absolute values of gait speed are comparable to published normal ranges in clinical settings. This study highlights the potential use of mobile accelerometry to assess gait parameters which may be indicative of future health outcomes in healthy individuals.

## Introduction

Walking speed is a fundamental parameter of human motion and is increasingly considered as an important indicator of individuals' health status [1]–[3]. It is a universal yet easily understandable outcome measure, which has been shown to be meaningful for the prediction of future health outcomes [4] and the assessment of health status in chronic disabling conditions [5]–[7]. Patients who exhibit “accelerated ageing” in diseases such as multiple sclerosis (MS) have reported loss of walking ability as their greatest fear [8]. Recently, the limitations of widely used outcome measures for walking ability in MS (Expanded Disability Status Scale [EDSS], Multiple Sclerosis Functional Composite [MSFC]) have highlighted the need to find more appropriate outcome measures [9], [10]. A valid outcome measure should combine specific expert knowledge, feasible methods of assessment, ability to discriminate among healthy and diseased individuals and, of course, have a significant impact on human health [11]. Walking speed has emerged as an interesting candidate in various diseases e.g. chronic obstructive pulmonary disease (COPD), MS, Parkinson's disease and cardiovascular diseases [12]. Walking speed has been associated with fall risk, mortality, mobility limitation and disability [13] and conditions which may not be apparent from routine clinical measures, e.g. subclinical atherosclerosis [4]. Moreover, walking speed was found to be the best physical performance measure for predicting the onset of functional dependence in a Japanese older population [14].

Short distance walking tests (e.g. 6 minute walking test, timed 25-foot walk [T25-FW]) are standard tools to evaluate walking speed in a clinical setting. Changes in walking speed as measured with the T25-FW have recently been accepted by the Food and Drug Administration (FDA) as an outcome measure in a phase III trial [15], but this method is far from being widely accepted. Major drawbacks are the time and effort needed, measurement error and high day-to-day variability [7]. A change of 0.1 m/sec and/or 10% is considered to be clinically meaningful [5], but the variability in a clinical setting in three repeated measurements is up to 20% [16]. More importantly, walking speeds in a clinical setting are unlikely to fully represent those in a free-living environment [17], typifying the fundamental problem of applying results from clinical trials to a real-world situation [18].

Accelerometry has been developed as a means to enable observer-independent, long-term assessment of activity-related parameters. Technical challenges in using body-worn ambulatory assessment methods are now being overcome, opening up the possibility for the collection of objective, prolonged physical activity records. There are many examples of accelerometer devices which work on generally the same principle but vary in design, technology, costs and sensitivity to motion [19]. One important challenge for researchers is to extract clinically meaningful parameters which may be used in the prediction of future disease outcomes [20].

Methods for predicting walking speed range from simple approaches which combine step length with frequency (i.e. pedometers), to more sophisticated algorithms using complex estimates based on biomechanical models of gait [21] and/or linear and non-linear models. Using mobile accelerometry as a tool to objectively measure and quantify walking speed and changes in walking speed in both clinical and free-living environments may help to determine normal ranges, critical thresholds [5] and establish walking speed as a useful outcome parameter in epidemiological studies and clinical trials [22].

Here we describe the use of a tri-axial, waist-mounted accelerometer (“actibelt” [23]) for the acquisition of physical activity data and the application of a novel, validated algorithm [24] to predict walking speed in free-living, healthy individuals. We investigated whether differences in several gait parameters existed between different age and BMI groups.

## Methods

### Ethics Statement

All participants gave full informed consent to take part in the study and ethical approval was obtained from the Cambridge 1 Local Research Ethics Committee (REC 09/H0304/42).

### Participants

A subsample of participants from the Cambridge CardioResource study was recruited for the assessment of physical activity using the actibelt. Cambridge CardioResource was a pilot study to determine the feasibility and acceptability of integrating research protocols within the framework of the UK's national blood service (NHS Blood and Transplant, NHSBT) (http://ceu.phpc.cam.ac.uk/research/cardioresource/). Blood donors were recruited, between February and September 2010, at community venues within a radius of approximately 60 miles from the Cambridge Blood Centre at Addenbrooke's Hospital (UK).

Inclusion criteria for routine blood donation are: age 17–65 y for first-time donors (repeat donors >65 y can continue to give blood provided they remain in good health), a minimum weight of 50 kg and general good health. Exclusion criteria for blood donation typically aim to protect either the donor or the patient receiving the donation from any harm. The full list of exclusion criteria can be found on the website of the UK National Blood Service (https://secure.blood.co.uk/c11_cant.asp).

Donors were considered eligible to take part in the study if they were fit to donate and fulfilled the normal criteria for donation. Donors who either chose to provide extra samples for other purposes, or who were first-time donors were excluded from recruitment.

### Collection of blood samples and physical measurements

In addition to the routine 470 ml blood donation, an additional (15 ml) blood sample was collected from consenting donors for the purposes of research. After the blood had been collected and participants had rested, measurements of height, weight and blood pressure were made by trained research staff. Height and weight were measured with participants wearing indoor clothing and shoes. Height was measured to the nearest 1 cm using a SECA Leicester portable height scale and weight to the nearest 0.1 kg using SECA 877 digital scales (SECA, Birmingham, UK). One reading of blood pressure and heart rate was taken using an OMRON M10IT (OMRON, Milton Keynes, Bucks, UK); the measurement was taken in the ‘non-donation’ arm and once the participant was seated comfortably and rested. Participants were also asked to complete a questionnaire, at home, covering medical history and lifestyle.

### Physical activity measurement protocol

At 12 sessions during 5 non-consecutive weeks between February and March 2010 donors were asked, at the end of their donation session, if they were willing to take home and wear the actibelt over a period of 7 days. Those willing to participate were given a short one-to-one tutorial on how to use the accelerometer (plus, supplementary information to take home) and instructed to start wearing the device the following morning. During the instruction phase, the accelerometer was switched on by a trained study researcher and donors were advised not to switch it off until the last day of their measurement. Additionally donors were provided with an activity diary and asked to record times when the device was removed and any activities conducted on each of the seven recording days. Subjects were asked to remove the accelerometer only while showering, taking a bath or swimming and (if they preferred to do so) during the night. Participants were advised to remove the device on the morning of the eighth day, switch it off and return it, in the post, to the study centre in Cambridge. If any problems were experienced using the actibelt, participants were able to contact the CardioResource team via the study telephone or e-mail help lines.

### actibelt

The actibelt is a tri-axial accelerometer with 100Hz sampling frequency; it has 512 megabytes of memory corresponding to 10 days continuous recording and a battery life >20 days [23], [25]. The accelerometer is placed inside a belt buckle which the wearer fixes around the waist by either a leather or elasticated belt. With this design, the device is discreet and unobtrusive and is located close to the subject's centre of mass. It can either be used for long-term monitoring in a free-living environment [26] (“week-in-a-box”) or activity assessment in a clinical setting (“rapid tests”).

### Data analysis

The following parameters were extracted from the raw accelerometry data collected on each day of wearing the device: adherence (i.e. amount of time during which the device was worn), the number of minutes spent in active and exercise mode, activity temperature (i.e. a measure for overall physical activity), coherence length (i.e. average length of uninterrupted periodic walking intervals), the number of walking and running steps, step frequency, walking speed based on both running and walking steps [24] and further parameters deduced from walking speed, i.e. the total distance travelled, the longest non-stop distance travelled and step length.

Subjects were excluded from the data analysis either if no demographic data was available or the belt was worn upside-down during any part of the recording period. Only days with an adherence of at least 6 hours were considered complete for analysis in order to reduce potential bias due to periods when the device was not worn. For statistical tests and linear models, parameters were averaged over the whole 7-day recording period.

In order to minimize the risk of publishing false positive results, the dataset was split into two separate and independent datasets: one for hypotheses generation (open dataset) and the second to test and validate the previously generated hypotheses (closed dataset) [27]. Basic exploratory analyses including boxplots and Spearman rank correlations were performed using the open dataset in order to identify the main hypotheses for validation. Subsequently, several gait parameters showing an association with age and BMI were selected for validation. Comparisons between the open and closed dataset were based on the Wilcoxon test for continuous variables and Chi-squared test for categorical variables. Additionally a linear model was fitted on the open dataset to further investigate the primary outcome. The primary outcome was considered validated if the slope of the closed linear model was between the upper and lower bounds of the 95% confidence interval of the open linear model (−0.0055 and −0.0016 m/s per year). Secondary outcomes were considered confirmed if the resulting p-value of the Spearman rank correlation test for the closed dataset was below 0.00625 (0.05/8 due to adjustment for multiple testing [28]). Pooling the open and closed dataset in order to obtain a higher number of cases after completion of the validation procedure was deliberately avoided.

Data were analysed using R software for statistical computing and graphics [29].

## Results

### Participants

**Figure 1:**
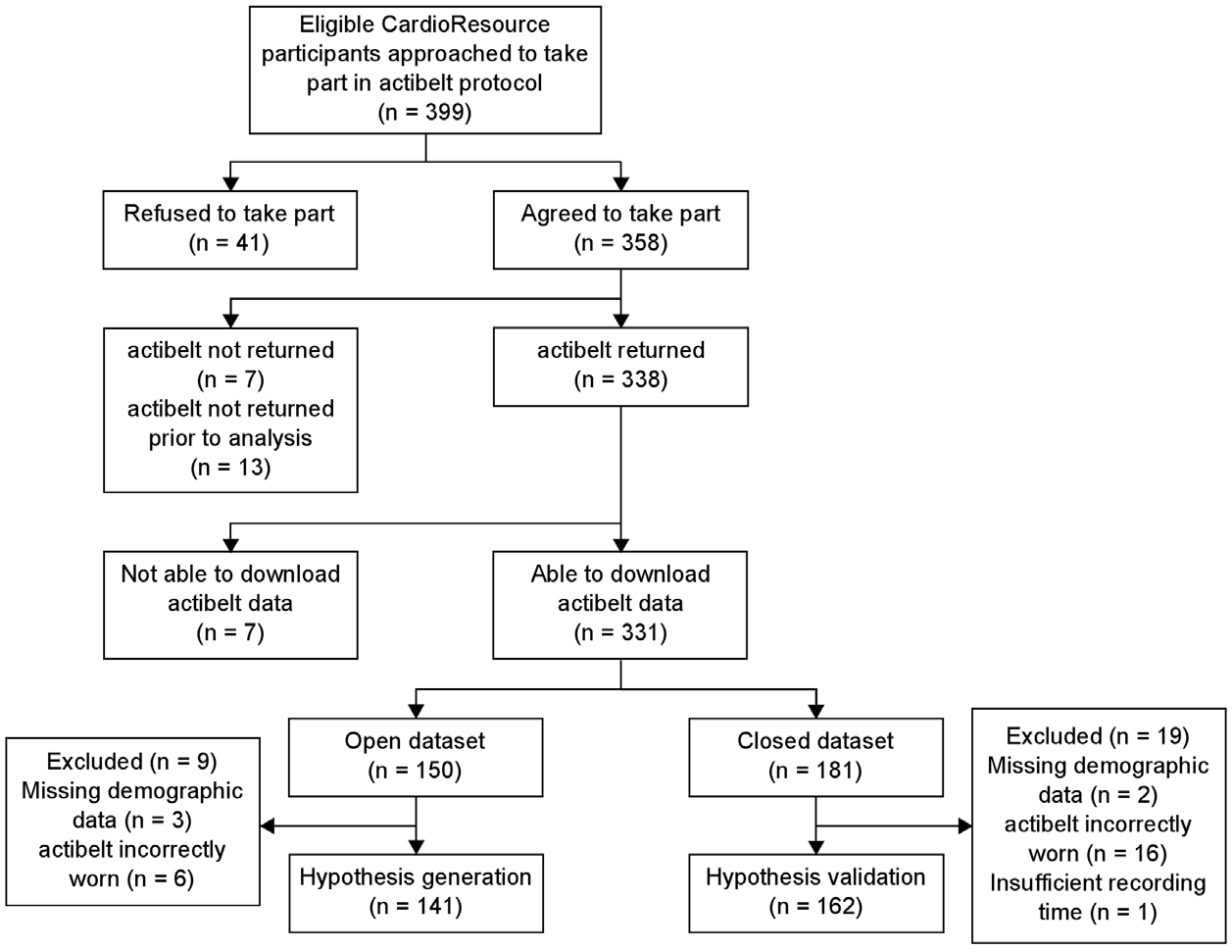
Flow diagram of participant involvement and division of data into ‘open’ and‘closed’ datasets.

**Figure 1** depicts the flow of participants from assessment of eligibility to the split between the open and closed datasets. The rate of uptake, response (i.e. return of actibelts) and successful download of data were 90%, 98% and 98% respectively.

**Table 1** summarizes participant characteristics in the open and closed datasets for whom demographic information was available. Although the open and closed dataset show statistical differences in the distribution between the sexes and median age, the populations are considered equivalent to answer the questions at hand.

**Table 1:** Participant characteristics (median [IQR]).

	Open	Closed	p-value
	*n = 141*	*n = 162*	
**Women, No. (%)**	72 (51)	64 (40)	0.04
**Age (y)**	52 (15)	48 (19)	0.03
**Height (cm)**	173 (12)	175 (12)	0.21
**Weight (kg)**	76.7 (19)	79.4 (21)	0.35
**BMI (kg/m^2^)**	26 (6)	26 (5)	0.91

### Hypothesis generation and validation

In the open dataset, three gait parameters showed significant correlations with age (**Table 2**) and four with BMI (**Table 3**). Of these associations, six were also significantly correlated in the closed dataset and confirmed by the validation process. The relationship between walking speed and age was selected as the primary outcome owing to existing evidence for the clinical relevance of walking speed both for the prediction of future health outcomes and as a novel, patient-oriented outcome measure, for the assessment of health status in chronic disabling conditions.

**Table 2:** Median (IQR) of gait parameters associated with age in open and closed datasets.

	All	Age (years)					p-value
		<30	30 - 39	40 - 49	50 - 59	>60	
**N**							
Open dataset	141	12	16	33	54	26	
Closed dataset	162	16	31	54	40	21	
**Walking speed (m/s)**							
Open dataset	1.25 (0.12)	1.34 (0.12)	1.26 (0.12)	1.26 (0.12)	1.23 (0.11)	1.21 (0.11)	<0.001
Closed dataset	1.27 (0.14)	1.27 (0.14)	1.27 (0.19)	1.29 (0.11)	1.28 (0.08)	1.20 (0.07)	0.004
**No. of running steps**							
Open dataset	65 (249)	534 (676)	112 (247)	157 (433)	47 (113)	26 (46)	<0.001
Closed dataset	103 (280)	122 (205)	176 (651)	164 (324)	87 (288)	24 (39)	<0.001
**Total distance (m)**							
Open dataset	6099 (3612)	8297 (1853)	7655 (2803)	6284 (4479)	5876 (3676)	5534 (2154)	0.001
Closed dataset	6226 (3777)	5425 (2818)	6624 (3291)	6004 (3769)	7621 (4633)	5622 (2860)	0.90

**Table 3:** Median (IQR) of gait parameters associated with BMI in open and closed datasets.

	All	BMI (kg/m^2^)			p-value
		<25	25–30	>30	
**N**					
Open dataset	141	56	61	24	
Closed dataset	162	66	66	60	
**No. of walking steps**					
Open dataset	8576 (4609)	9538 (4144)	8239 (3697)	5738 (3396)	<0.001
Closed dataset	8443 (5217)	9446 (4914)	8380 (5567)	6759 (3910)	0.002
**No. of running steps**					
Open dataset	65 (249)	116 (531)	47 (135)	32 (133)	<0.001
Closed dataset	103 (280)	117 (359)	124 (345)	23 (111)	<0.001
**Total distance (m)**					
Open dataset	6099 (3612)	7126 (3562)	5586 (2905)	4154 (2515)	<0.001
Closed dataset	6226 (3777)	6781 (3306)	6317 (4000)	4831 (2565)	0.002
**Longest n-s distance (m)**					
Open dataset	966 (864)	1376 (1453)	876 (635)	607 (569)	<0.001
Closed dataset	968 (969)	1168 (1190)	947 (808)	573 (581)	0.002

An additional linear model with age as the predictor and walking speed as the response variable was fitted on the open dataset in order to describe the relationship between those two variables in more detail. Intercept and slope in the open dataset were 1.4531 and -0.0037 m/s, respectively; in the closed dataset 1.3739 and −0.0020 m/s. Since the slope of the model when fitted on the closed dataset remained within the upper and lower bound of the 95% confidence interval (−0.0055 and -0.0016 m/s per year), the model was validated.

### Gait parameters and age

**Figure 2:**
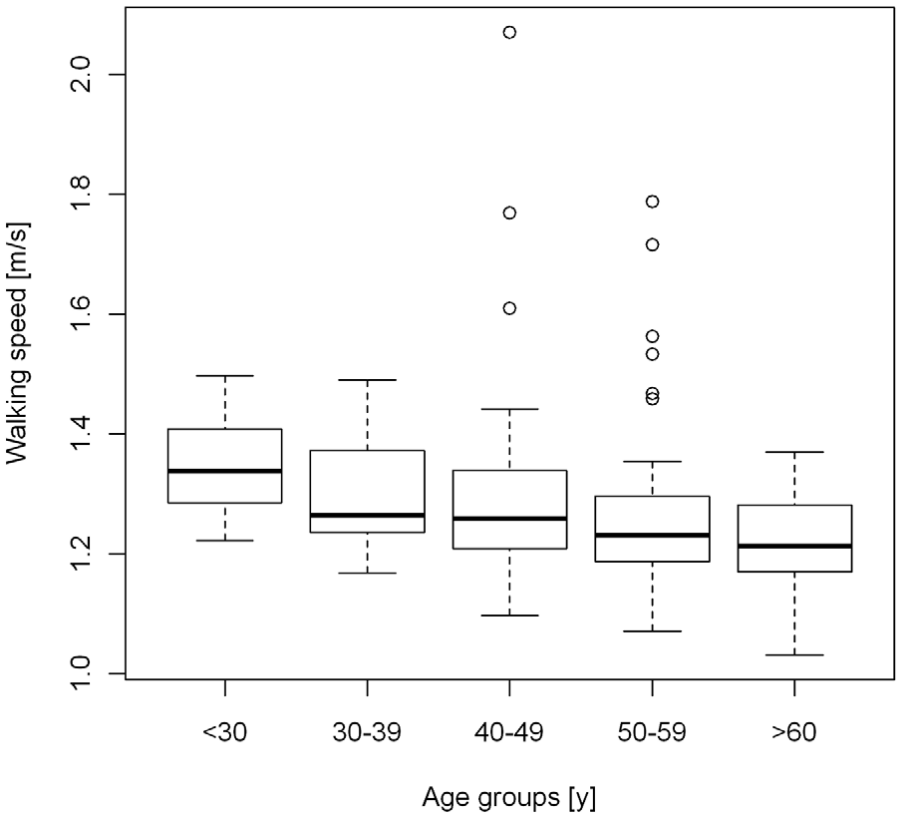
Boxplot showing relationship between walking speed and age.

Daily walking speed across all participants ranged from 1.03 m/s up to 2.07 m/s with a median walking speed of 1.25 m/s in the open dataset. **Figure 2** clearly shows the significant decline of walking speed with increasing age in the open dataset.

A post-hoc analysis was performed to further investigate whether reduction in gait speed with age was the result of fewer running steps with increasing age. However the relationship remained significant when running steps were not included in the calculation of gait speed (p-value: 0.0021).

Participants had a median (IQR) number of running steps of 65 (249) which were also shown to be negatively associated with age. The large number of running steps shown by one individual (i.e. 9953) was confirmed by reference to their completed physical activity diary in which the participant reported running for at least 30 minutes every day.

The association between age and total distance travelled (median 6099 m, IQR 3612 m) was only found in the open dataset and could not be validated in the closed dataset.

A Wilcoxon rank sum test (p-value: 0.13) did not suggest a significant difference in walking speed for male and female subjects. The effects of BMI and blood pressure, on walking speed were non-significant (p-values: 0.64 and 0.65, respectively).

For future applications of the methodology (e.g. in the context of clinical trials) it is important to also quantify the day-to-day variability of walking speed to determine the necessary number of observations per subject. A mixed-effects model with random intercept per subject was calculated to determine the day-to-day variability; this was found to be close to the effect size. The resulting standard deviation for day-to-day fluctuations is 0.14 m/s. Hence averaging weekly recordings would result in a variability of 0.053 m/s, about half the clinically relevant threshold of 0.1 m/s [5], [30].

### Gait parameters and BMI

There was a negative association between BMI and the number of walking and running steps and also the total and longest non-stop distance (**Table 3**). No differences in coherence length or step frequency were found between the different age and BMI groups.

## Discussion

To our knowledge this is the first study using accelerometry which shows an association between walking speed and age in free-living, healthy individuals. To date this has previously only been shown in a clinical setting [31]. Absolute values of gait speed are comparable to published normal ranges in clinical settings [6].

Walking speed has been shown to decline with increased risks for heart disease. In a group of ‘healthy’ participants, walking speed was shown to decrease with subclinical atherosclerosis [4]. While it is not possible to draw conclusions on the directionality of this association, one hypothesis suggested by the authors was that subclinical vascular factors may play a role in motor function as a result of increased cerebral white matter hyperintensities. If this hypothesis were true, then an association would be expected between blood pressure and walking speed, as hypertension is a main risk factor for white matter hyperintensities. However, Hamer et al. [4] and the authors of this study found no evidence of such an association.

The negative relationship between BMI and walking parameters (i.e., amount and distance) is consistent with the findings of Levine et al. [32]. In this study, free-living walking was measured using a system of multiple sensors integrated in specially designed body suit that recorded data on body posture and movement in lean and obese participants over a 10-day period of weight maintenance feeding and a 10-day period of overfeeding. It was found that lean individuals walked 3.5 miles/day more than obese individuals due to differences in the distance travelled, rather than number of walking bouts; this association may be attributed to the higher level of energy needed to walk a given distance with increasing BMI. With overfeeding walking declined similarly in both lean and obese participants; reduced levels of walking with increased weight gain may in turn lead to further weight gain due to concurrent reductions in energy expenditure and this may be reinforced through a continuous feedback loop.

A decrease in distances walked with advancing age has been self-reported [33] and demonstrated using the 6-minute walking test [34]. However, in this study, although a similar finding was shown in the open dataset, this was not replicated in the closed dataset.

A potential limitation of this study is the involvement of participants not drawn from the general population but from a select group of individuals who volunteer and meet the eligibility criteria for donating blood. However blood donors represent a diverse population group in terms of age, physical characteristics and lifestyle, which should allow extrapolation to a larger population group.

An additional consideration is that, for logistical reasons, the actibelt was worn for 7-days post-donation and the effect of blood loss on usual physical activity levels is unknown. There is no evidence that supports advice to avoid usual activities more than a few hours after having given blood, but it is unknown if and how much blood donation influences e.g. walking speed and the distribution of longest non-stop walking distance in the days after blood donation. There is evidence that blood donation - typically corresponding to a loss of roughly 10% of the total blood volume needing several weeks to be replaced - is linked to a decrease in endurance capacity, physiologically explained by a decrease of the total number of erythrocytes that are responsible for continuous supply of oxygen to the slow-twitch muscles fibres [35]. We believe that the effect is rather small leading to a potential small bias towards lower speeds and smaller distances. This effect should be small or negligible for normal persons with a lifestyle that is not highly active and when focussing on changes in time. Further studies are needed to quantify this potential source of bias for estimating absolute values of walking speed, but the key finding about a linear decline of walking speed in time should not be influenced.

Key strengths of this study include the high response and acceptance rates of the actibelt protocol. Very few helpline queries were received in relation to the use of the actibelts indicating that they were easy to use. Furthermore, in the data analysis, the authors have appropriately corrected for multiple testing and taken care to minimise the risks of reporting false-positive results by separating the exploratory analysis from the validation analysis [27], [36]. It is due to this careful validation policy that the authors believe that the association between walking speed and age is indeed true and not an artefact due to bias and confounding by unobserved heterogeneities such as social status, employment or environmental influences. The association between walking speed and age - which does not necessarily prove a causal relationship - showed a high degree of correlation in the open dataset which was subsequently validated in the independent closed dataset.

In addition, the association between walking speed and age is robust which was further tested by raising the threshold for minimum daily adherence from 6 to 10 hours; this did only have a negligible effect on the overall result and the association remained significant. Algorithms to automatically extract clinically meaningful parameters have been carefully validated in independent datasets [24]. Furthermore, unprocessed raw data for each participant was recorded and stored, so that future analysis with new algorithms can be performed if necessary.

In conclusion, this is the first study using accelerometry which shows an association between walking speed and age in free-living, healthy individuals. This study highlights the potential use of accelerometry, for both research and clinical purposes, to assess gait parameters which may be indicative of future health outcomes or changes in health status with lifestyle or medical interventions.
